# Influence of Liquid-to-Gas Ratio on the Syngas Fermentation Efficiency: An Experimental Approach

**DOI:** 10.3390/bioengineering7040138

**Published:** 2020-10-31

**Authors:** Spyridon Achinas, Sytse Jelmer Mulder, Gerrit Jan Willem Euverink

**Affiliations:** Faculty of Science and Engineering, University of Groningen, 9747 AG Groningen, The Netherlands; s.j.mulder.2@student.rug.nl (S.J.M.); g.j.w.euverink@rug.nl (G.J.W.E.)

**Keywords:** syngas, biomethane, methanogens, fermentation, biochemistry

## Abstract

Syngas fermentation by methanogens is a novel process to purify biogas. Methanogens are able to ferment non-desirable CO_2_, H_2,_ and CO to methane. However, to use methanogens on an industrial scale, more research has to be done. There are studies that discuss the growth of methanogens on syngas in combination with acetate. In this research, growth of methanogens on syngas as sole carbon source is discussed. Effluent of an anaerobic fed-batch was selectively cultivated with syngas in 400 mL Eppendorf© bioreactors. After a period of 7 days, fifteen 120 mL flasks were filled with three different liquid-to-gas ratios (1:1, 1:3, 1:5). Results showed that different liquid-to-gas ratios change the metabolic preference of the anaerobic microbial community. Moreover, complete conversion in a four-to-eight-day period, via the carboxidotrophic pathway, was observed in all three liquid-to-gas ratios.

## 1. Introduction

Syngas fermentation has been seen as a way to capture the excess of renewable produced energy and as an ex-situ manner to purify biogas production [1]. H_2_ electrolyzing and gasification of biomass could precede the ex-situ purification. Renewable produced electricity is in this case used to electrolyze H_2_ from water, also known as a power-to-gas solution [1,2]. However, H_2_ is difficult to transport in the current natural gas grid. On the other side, gasification of carbon sources results in the production biogas and syngas [3]. In order to use and transport the produced biogas through the natural gas grid, it has to be purified [4]. Both H_2_ and syngas can be used as organic feedstocks for ex-situ methanation. Hence, syngas by methanogens is a solution that uses a separated fermentation process. This separated fermentation process includes mainly methanogens that convert the non-desirable CO_2_, H_2,_ and CO concentrations within the anaerobic fermented biogas. It is an additional purification step to increase CH_4_ concentrations up to 99 mol% CH_4_ [4].

To be able to apply syngas fermentation on an industrial scale, more research has to be done about methanogenic growth [5]. Although around 150 methanogenic species are characterized, only four are able to grow on CO as a pure carbon source: *Methanobacterium thermoautotrophicum, Methanosarcani acetivorans, Methanosarcina barkeri,* and *Methanothermobacter marburgensis* [6,7]. Since CO is seen as an inhibitor to the growth of methanogens, it is interesting to show the ability of a mixed culture to grow on a medium with CO as primary carbon. It is known that methanogens have four carbonic metabolic pathways: carboxidotrophic, hydrogenotrophic, methylotrophic, and acetoclastic [8,9,10]. The four pathways are depicted in Equations (1)–(4). The ability to grow on a medium with CO as primary carbon source shows the ability of the anaerobic microbial community to use these pathways. The amount of anaerobic microbial community present (i.e., liquid) in relation to the gaseous feedstock present hypothetically influences the preference for a specific pathway. In this case, a specific liquid-to-gas ratio could optimize the industrial application of ex-situ anaerobic syngas fermentation [1,11].

4 CO + 2 H_2_O → CH_4_ + 3 CO_2_ (1)

4 H_2_ + CO_2_ → CH_4_ + 2 H_2_O (2)

CH_3_COH + H_2_ → CH_4_ + H_2_O (3)

CH_3_COOH→ CH_4_ + CO_2_ (4)

In this study, the methanogenic growth of an anaerobic consortium is studied for three different liquid-to-gas ratios. Interestingly, in all three ratio’s the concentration of CO is completely converted within eight days. Although similar CO conversion rates are found [3,7,12], these studies research the conversion of syngas to acetate or they use additional carbon sources for methanation. In this study, the major carbon source is syngas, which is used to produce methane. It is shown that a higher liquid-to-gas ratio increases the conversion rate and results in a higher metabolic preference for volatile fatty acids (VFA). In all ratios, both the carboxidotrophic and hydrogenotrophic pathways are used by the methanogenic community to achieve this conversion rate.

## 2. Materials and Methods

### 2.1. Materials

The anaerobic culture for syngas fermentation was obtained from a mesophilic digester that anaerobically treats wastewater at the wastewater treatment plant (WWTP) of Garmerwolde in Groningen, the Netherlands. The fresh inoculum used in the batch tests was selectively degassed to ensure anaerobic conditions and to keep methanogenic bacteria active. A two-liter glass bottle, containing 1.4 L fresh inoculum, was flushed with syngas for 10 min and placed in the incubator at 36 °C for 1 week. For the selective degassing, Stam medium was used to reinforce the methanogenic activity. The Stam medium included 0.265 g Na_2_HPO_4_·2 H_2_O, 0.205 g KH_2_PO_4_, 0.15 g NH_4_Cl, 0.055 g CaCl_2_·2H_2_O, 0.05 MgCl_2_·6H_2_O, 0.15g NaCl, 2.0 g NaHCO_3_, and 0.24 g Na_2_S·9H_2_O per liter (including 1 mL·L^−1^ acid trace element solution, 1 mL·L^−1^ alkaline trace element solution, and 0.2 mL·L^−1^ vitamins solution [13]).

### 2.2. Batch Tests

Fifteen 120 mL glass flasks were filled with inoculum and syngas with different liquid–gas ratios (working volume/headspace volume) and tested in mesophilic and thermophilic conditions. The flasks were filled such that three different ratios were made: a ratio of 1:5 (20 mL of liquid and 100 mL of gas), 1:3 (30 mL of consortium and 90 mL of gas), and 1:1 (60 mL of consortium and 60 mL of gas). The glass flasks were sealed and flushed with syngas (10% CH_4_, 10% CO_2_, 25% CO, and 55% H_2_) for 5 min to saturate the glass bottle and to ensure anaerobic conditions. This specific syngas composition was chosen because it is a composition that is acquired by gasifying biomass (i.e., natural carbon sources such as fossil fuels or agricultural waste). Thereafter, they were placed in an incubator, maintained at a constant temperature (35 ± 1 °C), and shaken manually twice per day during the experimental period of the assays. At day 4, day 6, and day 8 (end), a triplicate was removed, and their volumetric composition of the gas was measured. For every ratio and every time intervals, two control groups were added: (A) containing water and syngas and (B) containing the inoculum. Control A shows the uptake of the gaseous substrates in water at the specific day and control B shows whether the consortium produces methane without the addition of a syngas. Controls were used to correct the methane production from syngas.

### 2.3. Anlaytical Methods

The volumetric percentages of CO, CO_2_, and CH_4_ were measured on a gas chromatograph HP 5890 series II with a thermal conductivity detector. The carrier gas used was helium. The temperature of the column was set to 40 °C, after which the gas temperature was increased to 90 °C in 3 min. The detector was maintained at 90°C for 8 min. As a reference, a syngas mixture with a composition of 10% CH_4_, 10% CO_2_, 25% CO, and 55% H_2_ was used. The gasses were injected via a syringe at atmospheric pressure and room temperature. The HACH AT1000 automatic titrator was used to determine the total alkalinity (TA), the volatile fatty acids (VFA), and the pH of the samples. HACH © AT1000 automatic titrator uses the Nordmann method to determine these values. A 0.1 M of sulphuric acid solution was titrated to express the TA value in mg/L of calcium carbonate at a pH up to 5. After a second titration, the VFA value was measured in mg/L acetic acid between a pH of 4.4 and 5.

## 3. Results

### 3.1. Methane Production

**Figure 1:**
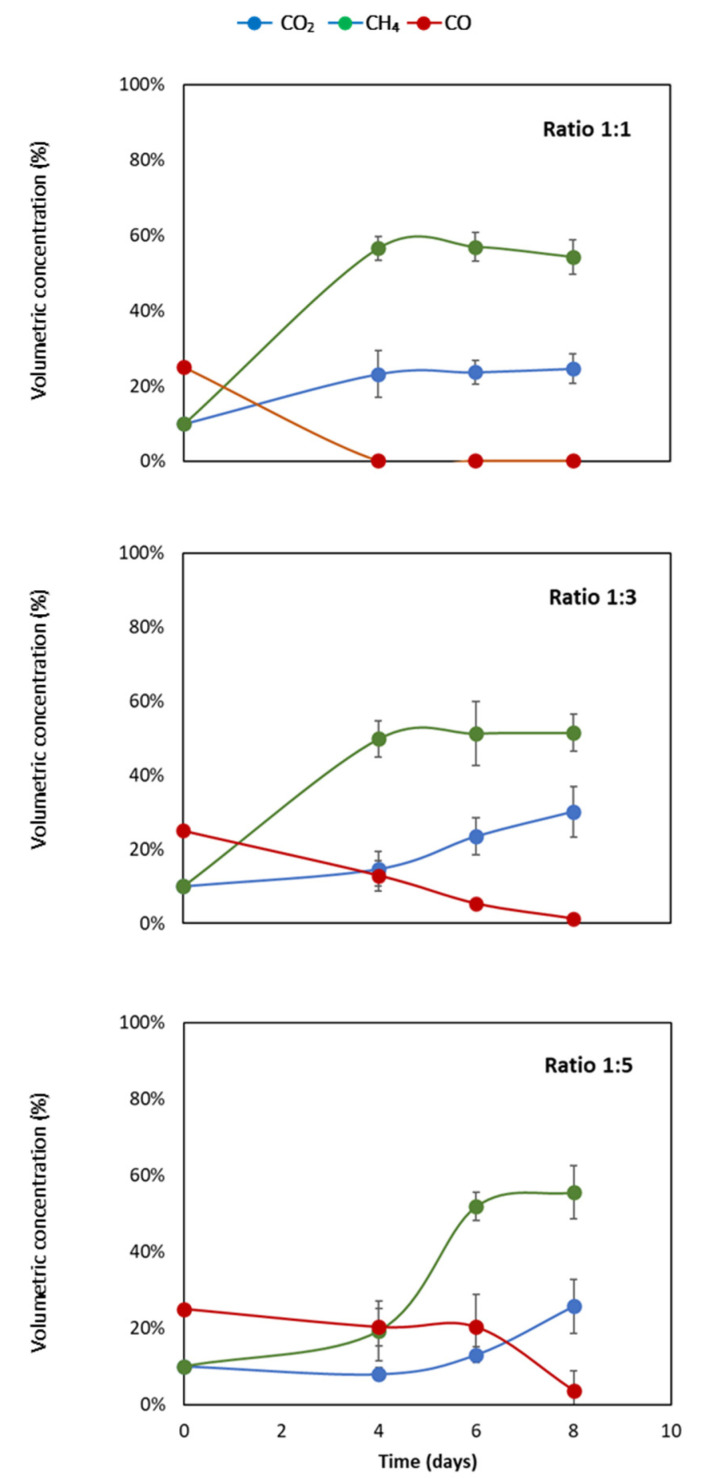
Volumetric composition of CO_2_, CO, and CH_4_ of liquid-to-gas volume ratios of 1:5, 1:3, and 1:1. The error bars represent the standard deviations of the triplicate samples. All data points contain error bars.

In Figure 1, the volumetric concentrations of CO_2_, CH_4,_ and CO in a 120 mL flask with three different liquid-to-gas ratios at 35 °C are presented. In ratio 1:1, the CH_4_ concentration increased from the initial 10 to 60% at day 4. After day 4, the concentration stayed relatively flat around a volumetric concentration at 60%.

The volumetric concentration of CO_2_ also increased, by 12%, from the initial 10% to 22% at day 4. Lastly, the volumetric concentration of CO decreased from 25% to 0% at day 4. The concentration of both CO_2_ and CO stayed flat around the concentration of, respectively, 22% and 0% at days 6 and 8.

In the liquid-to-gas ratio of 1:3, a gradual increase of CH_4_ was observed. The concentration increased from 10% to 50% at day 4 and continued to increase to 55% and 56% at days 6 and 8, respectively. At day 4, the volumetric concentration of CO_2_ in the headspace was measured to be close to the initial concentration of 10%. After day 4, the concentration increased from 15% to 25% on day 6 and day 8, respectively, as depicted in Figure 1.

Lastly, the volumetric concentration of CO decreased from 25% to 10% at day 4, and a further decline of the concentration was observed at day 6. Eventually, the CO concentration reached 0% at day 8. In the flasks with ratio of 1:5, an increase of CH_4_ concentration of 15% was observed in the first four days. At days 6 and 8, the concentration reached up to 56% and 58%, respectively. Moreover, the CO_2_ concentration stayed almost flat the first four days and afterwards increased to 18% and 25% on days 6 and 8, respectively. A change in CO concentration was observed at day 8, where CO decreased from 21% (day 6) to 3% (day 8).

### 3.2. TA, VFA and pH

**Figure 2:**
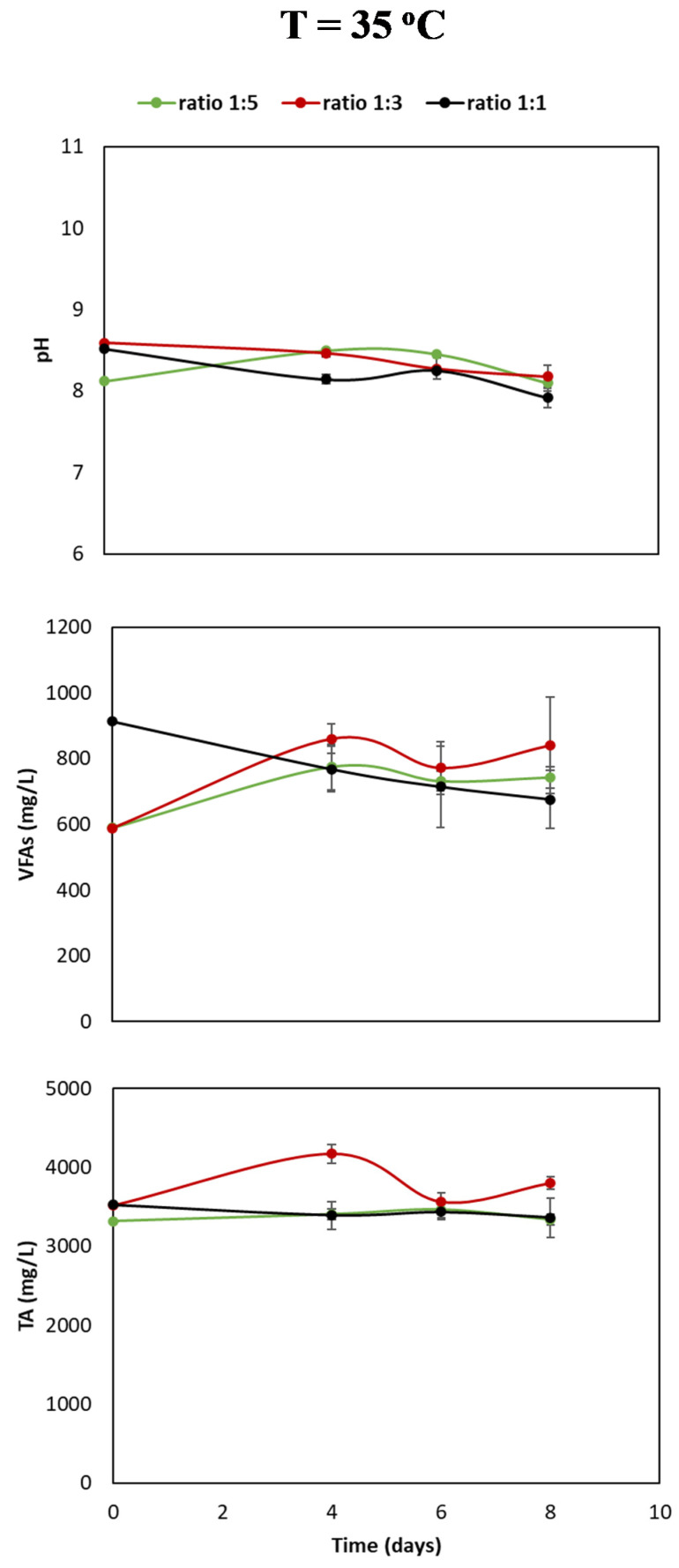
Change in buffer capacity or total alkalinity (TA), volatile fatty acid composition (VFA), and pH at three different liquid-to-gas ratios as a function of time (days). The error bars represent the standard deviations of the triplicate samples. All data points contain error bars.

In Figure 2, the changes of TA, VFA, and pH as a function of time (days) at different liquid-to-gas ratios are presented. TA values for the liquid-to-gas ratios 1:1 and 1:5 remained relatively stable over eight days (range 3150–3400 mg/L); however, at a liquid-to-volume ratio of 1:3, an increasing and decreasing trend could be observed, and TA value increased to 4000 mg/L on day 4 and then decreased on days 6 and 8 to 3900 mg/L.

The TA values of the ratio 1:3 were above the other ratios with the largest difference being approximately 550 mg/L, measured at day four. Furthermore, VFA present in the three ratios was determined. The VFA values in ratios 1:3 and 1:5 increased from an initial value of approximately 600 mg/L to values around 750 and 825 mg/L in eight days. The initial VFA value at gas-to-liquid ratio 1:1 was approximately 900 mg/L. In a period of eight days, it decreased to 700 mg/L. Lastly, the change of pH values after a function of time has been presented. In all three liquid-to-gas volume ratios, the pH value varied from 8 to 8.5 as a function of time irrespective of the volume ratio of liquid-to-gas. No significant increasing or decreasing trend was seen over a period of eight days. Thus, the archaea methanogens in the microbial community use hydrogenotrophic and carboxidotrophic metabolic pathways and therefore metabolize CO_2_ and CO, respectively, to CH_4_.

Based on Figure 1 and Figure 2, three main observations can be made: (1) a higher liquid-to-gas ratio increases the production rate of CH_4_; (2) in a higher liquid-to-gas ratio, CO is completely consumed in a period of four days; and (3) at a ratio of 1:1, the VFA increased, and in the other ratios the VFA decreased.

## 4. Discussion

### 4.1. CH_4_ Production by Methanogens

A significant increase in CH_4_ concentration can be seen in all ratios. In all the ratio experiments, the production yield of CH_4_ approaches 100% of the theoretical yield. The maximal theoretical volumetric concentration of CH_4_ is 57%. In the liquid-to-gas ratio of 1:1, a volumetric concentration of 60% is already reached at day four. It takes eight days for the other ratios to reach a concentration of 60%. Therefore, a higher liquid-to-gas ratio increases the production rate of CH_4_.

The concentrations of CH_4_ in the headspace depicted in Figure 1 are similar to the CH_4_ production obtained in a previous study [14]. They use a slightly different syngas composition, namely, 48.4% H_2_, 26.4% CO_2_, 23.3% CO, and 1.9% CH_4_. The production is similar, because in a timeframe of eight days the CO is completely consumed and converted to CH_4_. The production of CH_4_ stabilizes after six days. Finally, after nine days, they achieve a methane conversion of 50% of the theoretical yield in mesophilic conditions. Here the similarities end, because in this study a theoretical yield of 100% is observed. The use of a liquid solid instead of solid biochar explains this difference. Moreover, the theoretical yield does not reach 100% because additional VFA are made [7,14]. Methanogenic activity of the microbial community is exposed to syngas during the selective cultivation. This exposure could be the reason for more methanogenic instead of acetogenic activity.

The results show methanation yields exceeding 100% the theoretical yield. The theoretical yield is calculated considering the gaseous substrate as sole carbon source. Methanation yields exceeding the theoretical calculated yields therefore indicate a minor concentration of other carboxylic substrates initially present in the sludge. This is also explained by the minor initial concentrations of VFA depicted in Figure 2, namely, 900 mg/L and 600 mg/L for, respectively, ratio 1:1 and ratios 1:2 and 1:5 [15].

### 4.2. CO Consumption by Methanogens

Interestingly, the CO concentration in the headspace of all three liquid-to-gas ratios is completely consumed in eight days. There is, however, a big difference in the speed at which the CO is consumed. At ratio 1:1, the CO is already consumed at day four, where at ratio 1:3 the CO concentration is still 20%. It is logical that in a higher liquid-to-gas ratio the consumption is faster, because relatively more methanogens are present. However, the fact that the CO is completely converted in such a short time period is surprising. In a previous study, a complete CO consumption at nine days was shown, but not at four days [14]. Another study showed a complete CO consumption at 10 days [16]. Both studies exhibited similar mesophilic conditions and a headspace containing a volumetric CO concentration of 23.3% and 40% CO, respectively.

On the other hand, even faster consumption rates are presented in [6]. They prove that in thermophilic conditions, CO concentrations can be consumed in one day by a coculture of *M. thermoautotrophicus* and *Carboxydothermus hydrogenoformans*. However, these are thermophilic conditions that have been selectively cultivated. Moreover, a pure culture of the *M. thermoautotrophicus* completely converted CO in approximately eight days, although both were performed at different temperatures, which is in line with our study [6].

It is interesting to see a minor increase in the CO_2_ concentration. This indicates that the carboxidotrophic pathway is a preferred pathway by this methanogenic community. It is known that the carboxidotrophic and hydrogenotrophic metabolic pathways simultaneously convert CH_4_. It is also logical that the carboxidotrophic pathway is preferred, because it is thermodynamically more favorable than the hydrogenotrophic pathway [17]. The carboxidotrophic and hydrogenotrophic reactions are depicted in Equations (1) and (2).

### 4.3. Effect of VFA on Syngas Fermentation

In the ratio experiment, an increase in VFA values was observed as a function of time liquid-to-gas volume ratios of 1:3 and 1:5, see Figure 2. On the other hand, the VFA values of the experiment with a liquid-to-gas volume ratio of 1:1 decreased as a function of time. The decrease in VFA values can be explained by the variety of microbes present in the mixed microbial culture. Several species of this mixed culture are able to use formate, methanol, or acetate as catabolic product [18]. The production of CH_4_ from CO occurs along three routs: directly, via the hydrogenotrophic pathway, and by using acetate as intermediate [10].

The direct pathway, known as the carboxidotrophic pathway, can be seen in Equation (1). For the other two routes—via the hydrogenotrophic pathway and by using acetate as intermediate—methanogens need other anaerobic microorganic species. These species first convert CO to CO_2_ or acetate. However, they do not have the ability to further convert the CO_2_ and acetate to CH_4_. CO_2_ and acetate can therefore be seen as ‘’intermediate’’ products. Now it can be explained why the VFA values fluctuate at liquid-to-gas volume ratios of 1:3 and 1:5 as a function of time. The methanogens consume acetate as catabolic products to produce CO_2_, following the metabolic pathway of Equation (4), thereby reducing the VFA value as observed in the instance of a liquid-to-gas volume ratio of 1:1. On the other hand, in liquid-to-gas ratio 1:3 and 1:5, other microorganism present in the mixed culture use the CO and CO_2_ to produce acetate. The production of acetate increases the VFA value, as observed in the instance of a liquid-to-gas volume ratio of 1:1.

In addition, there is a reaction that will definitely be used by anaerobic microorganisms. This reaction is called the water gas shift reaction, see Equation (5). This reaction balances the concentrations of CO and CO_2_. It is therefore an important intermediate reaction for syngas fermentation by methanogens [9].

CO + H_2_O → CO_2_ + H_2_ (5)

A previous study found that a reduction of the VFA value by 47.8% resulted in a CH_4_ yield of between 62% and 67% [15]. However, in this case acetate was the main carbon source, and the concentration of VFA was 1400 mg/L, almost twice the initial VFA value measured in the gas-to-liquid ratio of 1:1. Use of another carbon source, apart from syngas, is often discussed in literature [10,12,14]. The experiment on the effect of liquid-to-gas ratio on syngas fermentation is a unique experiment, because no additional carbon source has been added. Only a very limited amount of VFA was initially present. Both the production and consumption of the VFA by the microbial community shows the vitality of methanogens. The quick depletion of the carbon sources present in the syngas changes the adaptation of the microbial community in the gas-to-liquid ratio 1:1. When relatively more gaseous carbon is available, in ratio 1:5 and 1:3, the methanogens change their metabolic pathways and produce more VFA. When a higher liquid-to-gas ratio is present, the VFA are used as additional feedstock.

To conclude, there is methanogenic activity present in the sludge. Under mesophilic conditions, the consumption of CO is more efficient than recent studies presented, and it can be concluded that the carboxidotrophic pathway is preferred over the hydrogenotrophic pathway. This suggests the presence of methanogenic species, known to be able to use CO as metabolic feedstock for methanation, such as *Methanobacterium thermoautotrophicum, Methanosarcani acetivorans, Methanosarcina barkeri,* and *Methanothermobacter marburgensis.* A topic for future studies could be to further identify specific species and their methanogenic metabolic activity. Furthermore, the change in VFA concentration in the reaction medium is different across liquid-to-gas liquid ratios. In a high liquid-to-gas ratio, the microbial community is more likely to use the present VFA as feedstock. In lower liquid-to-gas ratios, it is likely to produce VFA and therefore store energy in the form of carbon sources. The hypothesis is partly accepted, because on one hand there is carboxidotrophic metabolic activity that reduces the CO. However, on the other hand, the CO_2_ concentration does not decrease. This is due to the production of CO_2_ by the carboxidotrophic pathways and because the H_2_ concentration limits further consumption of CO_2_.

## 5. Conclusions

The methanogens indeed produced methane, and the CO was significantly consumed. However, the concentration of CO_2_ does not significantly decrease. This fact can be contributed to a limited amount of H_2_ in the volumetric gas composition. In general, the microbial community present preferred to produce methane rather than acetate. Complete CO conversion via the carboxidotrophic pathway was observed experimentally at different liquid-to-gas ratios. The short, four-, and eight-day periods, in which this occurred, were remarkable. According to the literature, complete conversion under mesophilic conditions took nine to ten days. Moreover, the production and consumption of VFA were observed in the microbial sludge. A larger liquid-to-gas ratio made the anaerobic microbial community prefer VFA metabolism. In media with a smaller liquid-to-gas ratio, the anaerobic microbial community produced VFA.
